# En-bloc Laser Resection of Bladder Tumors: Where Are We Now?

**DOI:** 10.3390/jcm11123463

**Published:** 2022-06-16

**Authors:** Massimiliano Creta, Giuseppe Celentano, Gianluigi Califano, Roberto La Rocca, Nicola Longo

**Affiliations:** Department of Neurosciences, Reproductive Sciences and Odontostomatology, University of Naples Federico II, 80130 Naples, Italy; dr.giuseppecelentano@gmail.com (G.C.); gianl.califano2@gmail.com (G.C.); larocca@unina.it (R.L.R.); nicola.longo@unina.it (N.L.)

Transurethral resection of bladder tumors (TURBT) is a crucial procedure in the management of bladder cancer. The goals of TURBT are to make the correct diagnosis, sample the detrusor muscle for staging, and completely remove all visible lesions [1,2,3,4,5]. The quality of the resection strongly influences patient prognosis and overall treatment success. TURBT can be performed by either conventional fractioned or en-bloc techniques. Although it is still the gold standard, conventional TURBT using the incision and scatter technique has a number of potential drawbacks. For example, thermal damage to nearby tissue can lead to difficulty in the pathological evaluation of fragmented tissue, and tumor fragmentation with a high number of exfoliated cancer cells could lead to infield and outfield recurrences [1,2,3,4,5].

First introduced in 1997 by Kawada et al., en-bloc resection of bladder tumors (ERBT) has recently emerged as a promising alternative to conventional TURBT [6]. It involves complete tumor removal and avoids incision through the tumor (a no-touch technique), thus respecting the conventional principles of oncological surgery. Technically, the procedure may be performed with different approaches and energy sources, e.g., knife electrodes, modified J-loops, monopolar or bipolar electrocautery, water jets, or lasers. Laser ERBT involves the use of laser beams to dissect bladder lesions, freeing them from their base and the surrounding tissue. A variety of lasers have been used to perform ERBT, including thulium, holmium, and KTP lasers.

Currently, only few randomized controlled trials have been published comparing laser ERBT to conventional TURBT, with follow-up ranging from 12 to 36 months [1,2,3]. Overall, laser ERBT appears to be a safer procedure for bladder tumor resection. Indeed, observed intra-and perioperative advantages of laser ERBT include: lower overall complication rates; absent obturator nerve reflexes and a subsequent low incidence of bladder perforation due to the lack of electrical effect; lower rates of post-operative bladder irrigation and lower bladder irrigation times; shorter catheterization times and lengths of hospital stay; and higher rates of the immediate postoperative instillation of chemotherapy [1,2,3]. A further advantage of laser ERBT includes the potential to perform the procedure without the cessation of anti-platelet or anti-coagulant drugs. One study found higher operative times with laser ERBT mainly due to the higher precision of resection and to the longer time needed for the laser treatment of anterior wall large tumors [2].

Based on the results from the pathological examination of tumor specimens, laser ERBT fulfills the oncological criteria of optimized resection with low residual tumor rates and improved specimen quality. Indeed, it provides higher detrusor muscle sampling rates (a surrogate marker of TURBT quality), and a lower incidence of residual tumors at re-TURBT [3].

Unfortunately, little evidence exists comparing laser and electrical ERBT. In their multicenter European study, Kramer et al. demonstrate comparable outcomes in terms of operation times, irrigation times, and length of catheterization and hospital stay in patients undergoing electrical and laser ERBT [5]. Detrusor muscle sampling was reported in 96.2% and 100% of specimens following electrical and laser ERBT, respectively [5]. A statistically low incidence of conversion to conventional TURBT, as well a statistically—although not clinically—significant advantage in terms of hemoglobin drop was noted in in patients undergoing laser ERBT [5]. Statistically insignificant differences were noted in terms of operation times, irrigation times, and length of catheterization and hospital stay. Detrusor muscle sampling was reported in 96.2% and 100% of specimens following electrical and laser ERBT, respectively [5]. Statistically insignificant differences in terms of recurrence rates were noted at the 12-month follow-up [5]. From a technical point of view, the authors consider the ability to cut a precise line around the tumor and the better vision obtained during laser ERBT as an advantage primarily for larger tumors [5]. On the other hand, however, switching from ERBT to conventional TURBT is easier when an electrical device is already being used [5].

A more recent study comparing monopolar, bipolar, and thulium laser ERBT confirmed similar rates of detrusor muscle sampling in the specimens and significantly lower rates of the obturator nerve reflex [6]. Out of six conversions to conventional TURBT, bladder cancer was found on the anterior wall and dome in five cases, and in the proximity of the meatus in one case [6]. Therefore, given the high rate of conversion for lesions in the anterior wall, the authors suggest a preference for electrical energy in these cases to avoid the increased potential risk of changing instruments and the subsequent waste of surgical material [6].

Despite promising preliminary evidence regarding laser ERBT, a number of issues remain under debate and under investigation.

Tumor selection criteria are still unconfirmed. Although it is estimated that ERBT is not feasible for almost 30% of tumors due to size, morphology, and/or location, laser ERBT has been performed for tumors up to 4.5–5.5 cm in diameter and in virtually all locations throughout the bladder [3,4].

Additionally, the risks associated with prolonged operative times, mainly in older patients, should be carefully evaluated.

Although the thulium–yttrium–aluminum–garnet laser is considered the device of choice by some authors when performing ERBT, due to its minimal penetration depth and decreased peak power, the search for a more efficient laser to perform ERBT deserves further investigation [7].

Finally, although insignificant differences in terms of recurrence rate have been found by some authors, most studies are not able to find differences in recurrence-free survival as the length of follow-up is still suboptimal, and long-term follow-ups are awaited.

## Data Availability

Not applicable.
